# COVID-19 as a psychological contagion: A new Pandora’s box to close?

**DOI:** 10.1017/ice.2020.127

**Published:** 2020-04-13

**Authors:** Dua Azim, Sohail Kumar, Sundus Nasim, Taha Bin Arif, Deedar Nanjiani

**Affiliations:** Dow Medical College, Dow University of Health Sciences, Karachi, Pakistan

*To the Editor—*In light of the recent pandemic, it is normal for the health practitioners, researchers, and policy makers to concentrate primarily on the pathogen and biological threats to understand the pathophysiology involved and recommend steps for the prevention and containment of the disease. The media and public health generally focus on the biological and physical ramifications of pandemics. Under circumstances that threaten to one’s existence, mental health issues secondary to the primary challenge are often avoided. However, stable mental health is one of the keys to fighting this ongoing pandemic and to restoring a post-pandemic society.

The outbreak of coronavirus disease (COVID-19) was deemed a public health emergency of global significance by the World Health Organization on January 30, 2020. The ubiquity of fear and angst, resulting in irrationality among people amid infectious outbreaks, is not uncommon. Past tragedies have proven that mental health effects can persist longer and can have a much higher prevalence than the disease itself. In a pandemic, although uncertainty raises stress and anxiety levels in healthy individuals, it also aggravates the symptoms in those with preexisting mental disorders.^1,2^ This phenomenon has been especially true for COVID-19. In a study conducted in China during the initial outbreak of COVID-19, 53.8% of the participants graded the psychological effects secondary to the outbreak as moderate to severe.^3^ This scenario raises many questions regarding the factors adversely affecting mental health and potential ways to approach this problem. To better understand the mental implications of a pandemic, it is mandatory to recognize and address the feelings attached to it such as fear, anxiety, and anger.

Although isolation, quarantine, and proactive social distancing are essential components for successful management of the current pandemic,^4^ mobility restriction associated with it is a major concern. A recent article by Brooks et al^5^ elucidates the psychological impact of lack of liberty among those quarantined. The uncertainty of the future causes distress to the affected individuals. When coupled with the restriction of social interaction, diminishing financial capital, and the continuous need for attention and treatment, this suffering can make life a living nightmare. Given the significance of mobility issues and financial crisis amid the COVID-19 outbreak, governments, as well as the private sectors, should deal with these issues efficiently by providing necessities to the commoners in the state of quarantine. It is essential that the general population does not succumb to this outbreak but stays organized and restricts circulation as much as possible and always to the fewest number of individuals.

Internationally, stigma and resentment aimed at affected populations by other nations due to fear of infection deter cross-border trade have stirred further unrest. In the ongoing COVID-19 pandemic, many instances of xenophobic attitudes against people of Asian origin have been publicized. These range from refraining from sitting next to Asians on public transport to physical and verbal abuse. Such emotions can be exacerbated by pre-existing mental disorders, leading to an intensified rumination of illness contraction. Thus, combined efforts of the state, leaders, and health agencies are vital for fostering social cohesion that is essential to the prevention of prejudice associated with an outbreak.^6^

Regarding financial issues, the finance minister of Hesse state in Germany, Thomas Schaefer, committed suicide due to the falling economy during a prolonged countrywide lockdown.^7^ It is unfortunate and deeply disturbing to lose a life over the financial burden. This leads to another major concern regarding the economic impact associated with the epidemic and current quarantine. With the closure of industries and community services, many people face unemployment and financial distress, further fueling the negative emotions endured by many individuals.^8^ For example, the economic impact of the current epidemic in Japan has been drastic.^2^ Thus, people who have lower incomes and are rendered unemployed during quarantine might need extra support in such times of need. Governments can reduce the financial effects of isolation by halting tax payments or even by providing financial reimbursements.

Another issue that warrants immediate attention concerns the spread of dubious information regarding the factors facilitating virus transmission, the number of people infected, and the mortality rate, which has led to fear and confusion among people. Easy access to communication devices such as mobile phones and various social media sites has proven to be a major contributing factor to the spread of inaccurate information. In turn, such misinformation has led to fear, confusion, and aggression among people.^9^ A lack of certainty about becoming infected and infecting others, as well as uncertainty about death, can aggravate a dysphoric mental condition.^2^ Thus, it is important to ensure that those affected by the outbreak have a clear understanding of the situation and the disease. It is up to the media to provide authentic information and regular updates regarding COVID-19 and professionals in infectious disease to alleviate the doubts. Last but not least, special attention should be given to the mental health of our healthcare workers at the frontline of the pandemic fight.^10^

All of the aforementioned stressors deeply affect mental health and cause fear anxiety, stress, and paranoia; they also create global hysteria that must be dealt with proficiently. The psychological impact of quarantine is wide-ranging and is likely to prevail for an extended period. Although quarantine is essential during this pandemic, authorities should carefully control this situation and should provide people with necessities such as food, water, and medical supplies, as well as clear communications containing timely accurate updates regarding COVID-19 and constant reassurance. Currently, no such organization is working to enhance the psychological health of individuals during this outbreak. Governments and other authorities should assemble a task force consisting of psychiatrists, psychologists, and other mental healthcare workers to provide psychological first aid to the people, to advise the government in matters of psychiatric health issues, and to create policies to intervene in such situations. If such measures are not given priority, then at the end of this pandemic, nations will be dealing with massive psychological suffering.
